# Barriers and facilitators to the use of Hypercare APK app in hypertension management: a qualitative inquiry into highly educated participants’ experiences in Ilorin, Nigeria

**DOI:** 10.3389/fdgth.2026.1891831

**Published:** 2026-07-31

**Authors:** Monsurat O. Yakubu, Deborah T. Esan

**Affiliations:** 1 Faculty of Nursing Sciences, College of Health Sciences, Bowen University, Iwo, Nigeria; 2 Department of Basic Nursing Sciences, University of Ilorin Teaching Hospital, Ilorin, Nigeria

**Keywords:** adherence, Hypercare APK app, hypertension, mobile health, patient experience, self-management, tertiary hospitals

## Abstract

**Introduction:**

Mobile health (mHealth) interventions offer promising strategies to enhance hypertension self-management; however, their real-world effectiveness depends on user engagement and contextual suitability, particularly in resource-limited settings. Evidence on highly educated participants' experiences with locally developed apps, including Hypercare APK app, remains limited. This study explored the highly educated patients' experiences with the Hypercare APK app, focusing on perceived usefulness, barriers, facilitators, and opportunities for improving its integration into hypertension care.

**Methods:**

A qualitative descriptive study was conducted among 14 adults with hypertension who had used the Hypercare APK app for at least 6 months in a tertiary hospital in Ilorin, Nigeria. Participants were purposively sampled from a digital health implementation study. Data were collected through in-depth semi-structured interviews and thematic and organization of participants analysed using Colaizzi's thematic approach.

**Results:**

Four themes identified were perceived usefulness of the application, facilitators of engagement, barriers to sustained use, and recommendations for improvement. The participants reported that medication reminders, educational messages, and self-monitoring features from the Hypercare APK app enhanced adherence and increased health awareness. The ease of use of the Hypercare APK app and access to information from the app facilitated engagement. However, structural barriers including unreliable internet connectivity, cost of mobile data, electricity challenges, and the app incompatibility limited consistent use. The participants opined that user-related barriers such as limited digital literacy, language constraints, and privacy concerns also affected its seamless use. The participants emphasised the need for offline functionality, multilingual support, simplified design, and integration into routine clinical care.

**Discussion:**

The Hypercare APK app was perceived by the participants in the tertiary hospitals in Ilorin as a valuable tool for supporting hypertension self-management in low-resource settings. On transferability, individuals with low or non-educational attainment may experience difficulties in using the app owing to poorer digital literacy. Notwithstanding, integrating mHealth interventions into existing health systems and designing contextually adaptive features are essential to enhance sustained engagement and equitable access.

## Introduction

Hypertension remains a leading contributor to global morbidity and mortality, particularly in low- and middle-income countries where health systems are often overstretched (1, 2). It is a major risk factor for cardiovascular diseases, including stroke, heart failure, and renal impairment, especially when poorly controlled (3, 4). Despite the availability of effective treatment options, global control rates remain suboptimal, largely due to poor adherence to long-term therapy (5–7).

Effective hypertension management requires sustained adherence to medication, lifestyle modification, regular monitoring, and continuous engagement with a healthcare provider (8, 9). However, multiple factors, including forgetfulness, limited health literacy, financial constraints, and inadequate access to care, continue to hinder optimal adherence (10).

In recent years, mobile health (mHealth) interventions have emerged as promising tools for supporting chronic disease management (11, 12). These technologies enable medication reminders, self-monitoring, health education, and remote communication with healthcare providers (13). While evidence suggests that mHealth interventions can improve adherence and clinical outcomes, sustained engagement remains a challenge, particularly in low-resource settings (14, 15).

Importantly, the success of digital health interventions depends not only on technological functionality but also on users' experiences, perceptions, and contextual realities (16). Factors such as usability, cultural relevance, digital literacy, and infrastructural constraints significantly influence adoption and continued use (17).

Although previous studies have explored mHealth interventions in hypertension management, there is dearth of qualitative evidence on highly educated participants lived experiences with locally developed applications like the Hypercare APK app in Nigeria. Understanding these experiences will be essential for designing contextually appropriate and sustainable digital health solutions (1).

This study was therefore aimed at providing qualitative inquiry into participants' experiences to the barriers and facilitators of the use of Hypercare APK app in Hypertension Management in tertiary hospitals in Ilorin, Kwara State, Nigeria.

## Materials and methods

### Hypercare APK app description

Hypercare APK app is an android mobile application designed to support the monitoring and management of patients with hypertension and related cardiovascular conditions. The application facilitates direct communication between patients and their assigned healthcare providers, enabling continuous remote health monitoring outside the clinical setting. The app supports two distinct user roles, each with a tailored interface and corresponding access privileges. It allows registered patients under hypertensive care to log vitals, view health records, communicate with assigned healthcare provider and receive medication reminders. The healthcare provider or assigned clinical staff member can monitor patient vitals, manage appointments, communicate with patients and generate health records in portable document files (PDF).

All patient data is stored in Google Firebase Realtime Database, a cloud-based NoSQL database with industry-standard encryption in transit (TLS). User authentication is managed by through Firebase Authentication using email and password credentials and each patient record is uniquely identified by a Firebase-generated UID. Access to patient data is role-differentiated, patients can only view and update their own records whereas healthcare providers have read access to all registered patient vitals through the dashboard. Health update timestamps are fetched from an external NTP server (time.goog.e.com) to ensure the accuracy and temper-resistance of clinical timestamps.

The clinical features of the app include: (i) vital signs monitoring where patients can record and track vitals such as blood pressure (mmHg), heart rate/pulse (beats per minute), body weight (kg), height (m) and age (years). These values are stored with accurate timestamps using Network Time Protocol (NTP) synchronization to ensure data integrity; (ii) longitudinal health records in which all vital sign entries are stored chronologically, enhancing longitudinal health record for each patient. Nurses can access these records from the patient management dashboard as a PDF with a built-in PDF export function and print or saved for clinical documentation and handover purposes; and (iii) patient dashboard (Nurse view) where the nurse-facing dashboard presents a real-time overview of all registered participants, displaying their most recent recorded vitals, allowing healthcare provider to quickly identify patients that need urgent attention. Others are (iv) appointment scheduling in which the app enables nurses to schedule appointments for patients using a built-in date and timer picker. Schedules for appointments are stored in the cloud and are visible to the patient. Patients can also view next upcoming active appointment with a live countdown timer (hours and minutes remaining). The nurse dashboard can also display the total number of appointments and schedules for the current day; (v). Medication reminders in which the app delivers automated push notifications to support medication adherence. Such reminders are scheduled daily at two fixed times (morning dose at 8:00 daily and Evening dose at 6:00 p.m. daily) and persist across device restarts through a Boot Receiver mechanism; (vi) Patient-Nurse Messaging where the app with a real-time messaging system (delivered via Firebase) allows direct two-way communication between a patient and their healthcare provider. The app sends push notifications for incoming messages when the chat window is not in focus; and (vii) Health tips where the patient home screen of the app displays randomly selected health education tips sourced dynamically from the cloud database. These tips are refreshed periodically at every 10 min to provide varied health guidance relevant to the management of hypertension and general wellbeing. Some of daily health tips from the Hypercare APK app included: avoid taking unprescribed drugs; keep a diary to know situations when your BP rises, e.g., work, stress, lack of sleep; have a regular sleep pattern; take your medications at the same time every day; instead of making use of salt, use locust beans and spices like ginger and garlic in moderation; and avoid sedentary lifestyle that can worsen hypertension.

Before commencing the use of the app for the intervention purpose, the participants received a structured orientation session where topics like introduction of the app, installation, account registration, login procedures, navigation of the interface of the app, recording and monitoring of vitals, medication reminders features and their relevance/uses, access to educational contents and some technical challenges and troubleshooting procedures were taught. Participants were also provided with one-on-one hands-on practical session, a user guide each and contact information for technical support.

### Study setting

The study was conducted at the University of Ilorin Teaching Hospital, Ilorin, Nigeria, a tertiary healthcare facility providing specialised services for the management of chronic conditions, including hypertension. The hospital serves a diverse population drawn from both urban and rural communities across Kwara State and neighboring regions. The setting was purposively selected for its active involvement in cardiology care and its participation in digital health initiatives, making it suitable for examining the implementation and use of mobile health interventions in a typical resource-constrained Nigerian healthcare context.

### Participants

The current study is an off-shoot of a parent study. In the parent study, a total of 144 patients with hypertension were recruited from the University of Ilorin Teaching Hospital (UITH) and the Kwara State University Teaching Hospital (KWSUTH). A multistage sampling technique was used to allocate patients into the experimental group (*n* = 72) from UITH and control group (*n* = 72) from KWSUTH in a non-randomised controlled trial. The experimental group received the Hypercare APK app in addition to usual care, while the control group received only the usual care. The intervention was delivered over 6 months and assessed at baseline (pre-intervention stage), and post-intervention of 3 months (post-test 1), and 6 months (post-test 2). However, the current study was then conducted after the post-test 2 which included recruiting fourteen adults (≥18 years) with a confirmed diagnosis of hypertension who had participated in the experimental arm of the parent study (the test group) with the sole aim of evaluating the participants' experiences on the use of the Hypercare APK app, facilitators, barriers and suggestions for app improvement.

A purposive sampling strategy was employed to recruit participants with direct and sustained experience using the application. The inclusion criteria were:

(i) participants must have been diagnosed with hypertension;
(ii) must have used the Hypercare APK app for the 6 months;
(iii) willingness and ability to provide informed consent; and
(iv) free will to participate in the interview

Participants were recruited from cardiology and internal medicine outpatient clinics during routine follow-up visits. Eligible individuals were approached by the researcher, provided with study information, and invited to participate. No financial or material incentives were provided to participants to minimise the risk of coercion.

On reflexivity, the interview was conducted by the primary researcher who happens to be a registered nurse of 31 years of experience, a registered public health nurse, a nurse educator with Master of Science degrees in Nursing Science and a doctoral researcher with interest and passion for digital health interventions for the management of chronic diseases, training in qualitative research methods and experience in conducting health-related interviews. The interviewer had prior involvement with the Hypercare APK app as it was used during the parent study with no prior clinical relationship with the participants. All of these contributed to minimizing potential influence on the participants' responses. Furthermore, measures put in place to reduce bias included maintaining reflexive notes to document personal assumptions, perspectives and decisions during data collection and analyses, holding regular discussions with the research supervisor, the second author in this instance, to critically review interpretations, coding decision and thematic development. All this ensured that the findings were the views expressed by the participants rather than preconceptions of the researchers. Again, open-ended questions and neutral probing techniques were adopted to allow free expression by the participants on their experiences with the Hypercare APK app, whether positive or negative.

### Study design

This study employed a qualitative descriptive design to explore patients' experiences with the Hypercare APK app for self-management of hypertension. A qualitative approach was considered appropriate as it enables in-depth exploration of participants' perspectives, behaviours, and interactions with digital health technologies within real-world contexts. The study was conducted over 6 months (October, 2025 to March, 2026) as part of a broader digital health implementation project in which the Hypercare APK app was integrated into routine hypertension care. Only participants who had used the application for at least 6 months were included, ensuring sufficient exposure to generate informed and reflective accounts of their experiences.

### Interviews and data collection

Interviews were scheduled immediately after clinic consultations to minimise participant burden. The interview continued until there was data saturation as described by Onilude et al. (18). The data saturation was evaluated simultaneously with collection and analyses of data. Arising from each interview, transcripts were reviewed and coded to identify the emerging concepts and themes. By the 12th interview, it was discovered that there were no substantially new quotes and themes were identified because the responses from the participants were not substantially different from those of the participants that responded earlier. In order to ascertain thematic redundancies and absence of emergence of new insights, two additional interviews were conducted. The consistencies of findings across these final interviews alluded to the proof that adequate depth and breadth of data have been addressed.

Furthermore, prior to data collection, the primary researchers and research assistant underwent training which focused on the objectives of the study, principle of qualitative research, ethical conduct of the research, informed consent procedures, confidentiality, administration of the semi-structured interview guide, effective probing techniques, active listening skills and management of recordings from the interview. The questioning was done in a way that it was open-ended, avoiding leading questions and maintaining neutrality. The participants were encouraged to express themselves freely on their experiences with the Hypercare APK app. All of these were measures to ensure consistency and uniformity in the interviewing process. Mock interview sessions were held to familiarize the interviewers with the interview guide and standardize the process and techniques. Feedback, which was to improve consistency and minimize interviewer bias was upheld.

Data was collected through in-depth, semi-structured face-to-face interviews conducted by the primary researcher, with support from a trained research assistant as needed. Interviews were conducted in a private setting within the healthcare facility to ensure confidentiality and participant comfort, and each lasted approximately 30–45 min.

A researcher-developed interview guide was used to facilitate discussions. The guide was informed by a review of the literature on mobile health interventions for hypertension management and refined with input from qualitative research experts to enhance clarity, relevance, and comprehensiveness. The interview guide explored four key domains: (i) Experiences on using the Hypercare APK app; (ii) Facilitators of the use of the app; (iii) Barriers to utilization; and (iv) Recommendations for improving the app. The interview guide was pre-tested with two hypertensive patients who met the inclusion criteria but were not part of the main study. Feedback from the pre-test informed minor revisions to improve clarity, cultural appropriateness, and flow. Data from the pre-test were not included in the final analysis.

Interviews were conducted in English and were audio-recorded with participants' consent. Field notes were taken during and immediately after interviews to capture non-verbal cues, contextual details, and emerging reflections.

### Data analysis

Data analysis was conducted concurrently with data collection using a thematic analysis approach guided by Colaizzi's seven-step framework (19). Although the analytical framework of Colaizzi was originally developed for phenomenological inquiry, the current study adopted its systematic and transparent procedures to support the rigorous analysis of participants' accounts. The study was not conducted within a phenomenological tradition, nor did it seek to explore the essence of lived experience, rather, it used Colaizzi's stepwise approach as an analytical tool to facilitate the identification, organization, and pattern of interpretation within the data. In line with a qualitative descriptive design, the analysis in the current study remained focused on providing a comprehensive summary of participants' experiences with the Hypercare APK app while remaining close to their expressed perspectives and meaning. All interviews were transcribed verbatim and analysed using ATLAS.ti (version 25) (20). The analytical process involved (i) familiarisation with the data through repeated reading of transcripts; (ii) extraction of significant statements relevant to the research questions; (iii) development of initial codes through inductive analysis; (iv) organisation of codes into categories and sub-themes; (v) clustering of related categories into overarching themes; (vi) iterative review and refinement of themes through team discussions; and (vii) integration of findings with illustrative participant quotations.

To enhance analytical rigour, coding and theme development were reviewed through peer debriefing among members of the research team (21). An audit trail documenting coding decisions, theme development, and analytical reflections was maintained throughout the study.

Furthermore, trustworthiness was established through the criteria of credibility, dependability and transferability (22). Credibility was enhanced through prolonged engagement of the primary researcher and assistants with participants during the periods of data collection, audio-recorded interviews, verbatim transcription of the recorded interviews, repeated reading of the transcripts to ensure familiarity with the data, and the inclusion of direct participants quotations to support the findings. In addition, data collection and analysis were conducted iteratively in such a way to allow emerging issues to be used in subsequent interviews and to facilitate the assessment of data saturation. On the other hand, dependability was strengthened by the use of a semi-structured interview guide, adopting consistent data collection procedures and the maintenance of an audit trail that documented methodological decisions throughout the duration of the research. Furthermore, coding was performed independently by the two researchers in this study to enhance reliability. Emerging codes, categories, and themes were extensively discussed during regular meetings of the primary researcher and research supervisor. Any differences in the interpretation were resolved through discussion and consensus to ensure consistency in the analytical procedure (23). In addition, transcripts and preliminary interpretations were returned to the selected participants for verification and confirmation of accuracy of the contents (23). Confirmability was enhanced through reflexives practices and subsequent debriefing to minimize researcher bias. Finally, thorough detailed descriptions of the study setting, sampling procedures and data collection were yardstick adopted to enhance transferability. In this study, transferability should be considered in the light of the single-centered study and the highly educated participants.

### Ethical considerations

Ethical approval was obtained from the institutional Research Ethics Committee (Protocol ID: NHREC/02/05/2010). Written informed consent was obtained from all participants before data collection. Participants were informed of the study's purpose, procedures, potential risks and benefits, and their right to withdraw at any time without consequences for their clinical care. Confidentiality was ensured through anonymisation of transcripts using participant codes (e.g., P1, P2). All data were securely stored on password-protected devices accessible only to the research team. The study was conducted in accordance with the ethical principles outlined in the Declaration of Helsinki.

## Results

### Participant characteristics

Fourteen participants aged 23–55 years with confirmed diagnosis of hypertension were included in the study. The duration of hypertension ranged from less than one year to over 15 years. Participants had diverse educational backgrounds, ranging from Higher National Diplomas to postgraduate degrees, and were predominantly civil servants, lecturers, and business owners. Most participants were married. These relatively educated participants provided rich insights into user experiences with the Hypercare APK app. The demographic profile of participants is presented in Table 1.

**Table 1:** Demographic data of the participants.

ID	Gender	Age	Education level	Occupation	Marital status	Years with hypertension
P1	Male	55	Ph.D.	Lecturer	Married	>10 years
P2	Female	43	BSc	Civil servant (educationist)	Married	∼15 years
P3	Female	48	HND	Businesswoman	Married	<1 year
P4	Male	45	BSc	Civil servant	Divorced	∼7–8 years
P5	Female	23	Graduate	NYSC	Single	∼1.5–2 years
P6	Male	45	University (Distance Learning)	Student	Married	∼7 years
P7	Male	41	LL. B.	Private practice	Married	<1 year
P8	Male	33	Graduate	Civil servant	Married	∼1.5 years
P9	Female	33	Graduate	Civil servant	Married	∼2–3 years
P10	Female	55	HND	Civil servant	Married	∼10 years
P11	Female	31	BNSc	Nursing	Single	∼5 years
P12	Male	40	Graduate	Civil servant	Single	∼5 years
P13	Female	46	Ph.D.	Lecturer	Married	∼5 years
P14	Female	40	HND	Civil servant	Married	∼4 years

Ph.D., Doctor of Philosophy; BSc, Bachelor of Science; HND, Higher National Diploma; LL. B., Bachelor of Laws; BNSc, Bachelor of Nursing Science; NYSC, National Youth Service Corps.

### Themes

Arising from the participants, four themes were identified as perceived usefulness of the app, facilitators of engagement, barriers to sustained use, and recommendations for improvement (Table 2).

**Table 2:** Themes, sub-themes and codes for the responses.

Themes	Sub-themes	Codes
Experience with the use of the mobile app	Participants experiences	App effectiveness Ease of use Tracking/monitoring of vitals
Facilitators	App features	Daily education tips Information access Medication reminders
	App facilitating factors	Aids medication adherence Remote monitoring Support medication reminders
Barriers	Technology/resource barriers	Device compatibility Installation/Login issues Network issues
	Resource barriers	Data availability Electricity issues
	Security barriers	Concerns about privacy
	User's capacity barriers	Discomfort with reminder alert Lack of technical know-how Language barrier Forgetfulness
Suggestions for app improvement	Institutional support	Awareness about the app Encouragement from clinicians Government support
	Service integration	Incorporation into hypertension treatment Add doctor's appointment Broader health management
	Functional enhancement	Ease of set-up More user-friendly interface Multilingual support Offline functionality

### Experiences with the Hypercare APK app

On participants' experiences about the Hypercare APK app, majority of the participants perceived the app to be effective in supporting self-management of hypertension, tracking and monitoring health indicators particularly blood pressure whilst most participants described the app as simple enough and very easy to use. Majority of the participants emphasized the app was useful in supporting daily hypertension management. Three interrelated sub-themes emerged which were perceived effectiveness, ease of use, and support for self-monitoring.

### Perceived effectiveness

Participants consistently described the application as a practical tool for prompting to take hypertensive medications and lifestyle awareness. Reminder notifications and educational messages were particularly valued for reinforced treatment routines and increasing awareness of hypertension management (Table 2). A representative quote in this sub-theme from one of the participants (P1) is as follows:

“It keeps reminding you that you have hypertension… it helps you adhere strictly to your medication” (P1; Table 3).

**Table 3:** Qualitative expression of the participants on the various themes and sub-themes.

	Themes and sub-themes			
Participants ID	Participants experience	Facilitators	Barriers	Suggestions for app improvement
	Ease of use of app Tracking and monitoring of vitals App effectiveness	Aids medication adherence Remote monitoring Support for medication reminders	Technological barriers Resource barriers Security barriers User's capacity barriers	Institutional support Service integration Functionality enhancement
P1	*“as it is designed seems to have possessed all what is needed because I think the objective of the app is to keep reminding one that yes you have hypertension… so I think it's a very good app*”, and further emphasised that “*it has incorporated the most important future that can make one to adhere strictly to taking one's drug, which might then lead to reduction in the blood pressure*”,	“*there are instances where I almost forget to take my drug… but the moment the message comes in… it will give me a kind of prompting to go and take the drug*”.	“*an initial challenge… of trying to log in*”,	“*a fantastic innovation… for the management of hypertension… it can instil adherence to the drug regimen*” “*there should be… a policy that will involve incorporating this app into the healthcare delivery system*”,
P2	“*it's easy to read it and understand*”, “*I use it every day… to track my blood pressure… so that I will know whether it's increasing or reducing*”.	“*there are some particular daily tips… education tips that helps in managing lifestyle… and that education tips is something I look for every day*”.	“*the imputing of… email… password… if not because I am educated… it might be much more difficult for people that are not*”.	“*if it is incorporated in the healthcare facilities… it is a very good thing to do*”
P3	“*the app… reminds to go and take drug… how to monitor the blood pressure… it has benefits*” “*I use it… to monitor my blood pressure… it used to give me some information, remind me the time to take the drug… also, I need educated measure*”	“*the one that motivated me first is the reminder*”.	“*some people may not have Android… or they may not be able to operate Android… so that can be a major challenge*”. “*my email… first it didn't take it, but when I repeat doing it accepted it*”, “*it's not all of us understand English… they should try and input some local language*” that “*sometimes I could be busy… forgot to use it*”,	“*if they can introduce it in our hospitals in Nigeria… it will be beneficial to some hypertensive patients*”.
P4	“*with the use of the app… when the blood pressure increases, I will try and find what is causing that… and help to comply with the time of treatment*”.	“*apart from that too, you can easily read some educational content, messages that can help*”. “*…very, very useful… during this period that one is usually carried away and engaged in a lot of activities… by the time the reminder comes up… it assists somebody… to take his drug, to be careful of what you eat as well*”.	“*even when I remember, at times I may not have data… even when I have data, at times I will not even on my data*”.	“*there should be more enlightenment… if there is more enlightenment I think many people will embrace and they will appreciate it*”.
P5	“*I feel like the app has been most useful in monitoring my blood pressure… the fact that I have to input the values… my blood pressure and my weight*” “*my experience with it so far has been good… it's a very easy app to use… it doesn't require much stress*”. “*before I had the app I never really had any cause to monitor it… but with the use of the app… it has been helping me with monitoring it*”	“*I cannot forget the daily tips they have been so useful… almost every time try to rest well do this, do that*” “*there's always a reminder that comes up on my phone… go and use your drug… and it has been really helpful with me taking my drug*”,	“*we tried on my iPhone at first but it didn't support it… I had to use my Android phone because the app was not working on my iPhone*”. “*about 50% of the elderly… are not literate… this will limit them from using it*”.	“*there also has to be some form of enlightenment… because if I did not meet you now I wouldn't know anything about this app*”. “*maybe government can subsidise… especially Android phones*”,
P6	“*simple, very easy to input the data… and gives a constant reminder on management of hypertension*”.	“*So it helps one to see how you are doing. Maybe the BP is on the normal side*”	“*most people… are using these small phones… or touch light phones… they will not be able to use the app*”, “*at times I will not even remember*”,	“*awareness too can help… and the awareness will initiate a kind of reminder*”, “*the clinicians if they can encourage us… even forcing us to be using it… I think that would be an added advantage*”.
P7	“*it's not really user-friendly… I don't have a monitor at home… I only go once every month… I’ve been able to send two of my readings*”	“*And these are so informative. Honestly, I need to, I need to acknowledge that. Sometimes I just see them on my phone and I go through them. And of course, it will tell me that I should be using my drugs the same time every day to help*”	“*it's not constant… at times it will give the reminder… at times… there's no*”,	“*it's good to help a lot of people if it is incorporated into that system… you have to go nowhere to check what is affecting your health*”,
P8	“*from my own perspective, it's helpful… I will give it a 90%… for someone like me that do forget to use my drugs, the reminder, it helps… and there are other messages… like the health tips… it's really effective… it's a good help so I really love it*”,	“*even when I’m actually upset minded… it will remind me… and it helps me a lot to quickly switch back and take my drugs*”	“*my phone is indicating… it's a harmful app… initially I was actually scared*”,	“*it really, really helped to manage hypertension… I will be very happy if it can be incorporated*”.
P9	“*In fact, I like everything, but the one I like most is the reminders*”	“*the one I like most is the reminders*”.	“*that is data… that's the only obstacle that I face using it*”	“*if they can do it so that you will not be looking for data up and down before you can make use of it*”,
P10	“*When you get a reminder, go and do this I mean, common sense, you go and do something Like take your drugs I've taken your drugs, I've taken it twice That kind of a thing You go and take an action You know, that issue I think I prefer the reminder*”	“*I actually preferred the daily reminder… that reminds you to take your medication and also to give you educational messages about what you could do to manage your BP*”.	“*if you don't have data or if your phone is not charged… not that it's the app itself, but accessing it*”, “*the app is basically for somebody who is literate… if you understand only Yoruba, it doesn't work*” “*voice prompts in Yoruba or Igbo*”, “*if you’re in a meeting… and your phone is not on silent… it could be very, very embarrassing*”,	“*if it will be incorporated under different languages, you will have a wider reach… people who are illiterate are having a lot of issues dealing with their blood pressure*”.
P11	“*at least it helped me to check… to observe myself*”,	“*there was no obstacle not at all…the interface… is not difficult to operate*”.	“*I don't know… I couldn't operate it… maybe once weekly I don't know if it's on a daily basis*”,	“*the routine of going through the email address… password… is making it a bit stressful… if you just put your name… everybody can write their names*”.
P12	“*due to my work, I’m always busy at times… but the reminder always keeps me on*”.	“*one can sit down at home and access almost any information you need*”. “*instead of travelling… from nearby village to the hospital just for follow-up this can be a kind of follow-up*”.	“*the network has been a technical issue*”, “*you are not familiar with… just input your own personal data… there's kind of insecurity*”,	“*when it is introduced into the health sector, it's going to reduce the pressure on the health worker*”,
P13	“*the one I like most is the reminders*”.	“*when one is usually carried away… by the time the reminder comes up… it assists somebody… to take his drug*”	“*technical issues when I’m in the process of trying to use the app*”,	“*the interface be easy to access not having hitches*” “*making it more user-friendly*”,
P14	“*the time that I can say the app is most useful is when it reminds me to take my drugs… because sometimes I forget*”.	“*I like the daily education tips*”,	“*a bit sceptical about… inputting my email and password*” “*it's just the privacy and the cost of data*”.	“*once you download it, you can use it without data*”,

For many, the application addressed forgetfulness and competing daily priorities, acting as a behavioural prompt for medication-taking.

### Ease of use

Most participants perceived the Hypercare APK app as simple, intuitive, and easy to navigate, requiring minimal technical effort as expressed by a representative quote (P5).

“It’s very easy to use… it doesn’t require much stress” (P5; Table 3).

However, usability challenges were reported by a minority of participants, particularly those with limited access to home monitoring devices or lower digital confidence.

### Support for self-monitoring

The application enhanced participants' engagement in self-monitoring behaviours, particularly regular blood pressure tracking. Several participants reported increased awareness of their health status and more proactive management of their condition. This is expressed as a quote by P5 (Table 3).

“Before I had the app, I didn’t really monitor my blood pressure… now I check regularly” (P5).

This suggests that the application facilitated behavioural change by encouraging routine self-observation and accountability.

### Facilitators of app use

With respect to the second theme, app facilitating factors, majority of the participants mentioned how many times and consistently the reminders and daily education tips come into their phone supporting medication reminders and prompting them to take their medications (medication adherence). Participants consistently described it as essential in supporting adherence, particularly in the context of busy and unpredictable daily lives. Many spoke candidly about forgetfulness and how the app compensated for it. Whilst the daily education tips and information access was mentioned to enrich the understanding of users and supported lifestyle changes, it was the medication reminders that stood out as the most impactful, acting as a reliable prompt that bridges the gap between intention and action. However, remote monitoring as a component of facilitators was less frequently mentioned by the participants. Two key sub-themes that influenced sustained engagement were app features and perceived functional benefits.

### App features

Participants identified three core features that enhanced usability and engagement as (i) medication reminders which were the most frequently cited and impactful feature; (ii) daily education tips, providing actionable lifestyle guidance; and (iii) access to health information that enables independent learning. Reminder notifications were particularly influential in promoting the taking of medications promptly as reflected in the quote P1 (Table 3):

“When the reminder comes… it prompts me to take my medication immediately” (P1).

### Functional benefits

Beyond individual features, participants described broader benefits of the app to include: (i) regular prompting to take medication; (ii) enhancing the motivation for self-care management of hypertension; and (iii) potential for remote monitoring to reduce unnecessary hospital visits as opined by P14 (Table 3).

“It motivates me to take my drugs… it’s like a follow-up even when I’m not in the hospital” (P14).

These findings suggest that the Hypercare APK app functioned as both a behavioural support tool and a complementary extension of clinical care.

### Barriers to app use

On the theme of barriers to the effective use of the app, the participants hinted on a complex interplay of factors ranging from technological, through resources and security to user capacity. Some participants reported encountering compatibility issues, with the app not being compatible with their phones, particularly across different operating systems. Beyond compatibility, access to appropriate devices remained uneven. Some also reported initial technical difficulties, most especially with installation of the app and login of usernames and passwords. Some believed the app was complex with the issue of security risk and privacy concerns. Unstable network connectivity also posed challenges to the consistent use of the Hypercare APK app. According to the participants, the resource barrier focused on inadequate supply of and constant outages of electricity, as well as cost and availability of mobile data. The user's capacity identified by the participants include the fact that the Hypercare APK app has been conditioned in English language only. Some participants expressed difficulty navigating the app due to lack of familiarity with digital tools and discomfort with the constant reminder tips.

The quotes relating to each sub-themes are as expressed by representatives of the participants were grouped into four sub-themes of technological, resource-related, security-related, and user-related barriers.

### Technological barriers

“It didn’t work on my iPhone… I had to switch to Android” (P5; Table 3).

### Resource constraints

“Sometimes I don’t have data… or my phone is off because there is no electricity” (P8; Table 3).

### Security concerns

“I was a bit sceptical… you don’t know if it’s safe” (P10; Table 3).

### User capacity barriers

“If it’s only in English, some people will not be able to use it” (P10; Table 3).

### Recommendations for improvement

Many of the participants proffered practical solutions that can improve the app to include three sub-themes of Institutional Support (need for increased awareness and public enlightenment about the app, influential role of healthcare professionals in promoting the use of the app and integrating digital tools into existing patient-provider relationship and government to address economic and accessibility barriers like cost of the device, provision of constant electricity and reduction in the cost of data), Service Integration (integration of the Hypercare APK app into formal healthcare systems for hypertension management, desire for the Hypercare APK app to go beyond medication reminders by supporting continuity of care through appointment scheduling and expansion of the Hypercare APK app to support the management of other chronic conditions), and Functional Enhancements (ease of setting up the app, for the app to be more user friendly, multi-lingual support, simplified registration process and making the app to function in offline mode after installation). The integration of the app into the healthcare system is supported by this quote from P2:

“If it is incorporated into healthcare delivery… it will really help” (P2; Table 3).

These suggestions highlight the importance of contextual adaptation for sustained adoption in low-resource settings.

## Discussion

This study provides in-depth insights into highly educated patients' experiences with a locally developed mobile health application, Hypercare APK app for hypertension management in two tertiary hospitals (University of Ilorin Teaching Hospital and Kwara State University Teaching Hospital) in Ilorin, Nigeria. These tertiary health institutions were selected because of the proximity and ease of accessing participants. Overall, the findings indicate that the Hypercare APK app was perceived by participants as a useful, acceptable, and supportive tool for enhancing medication adherence and self-management. However, on transferability, individuals with low or non-educational attainment may experience difficulties in using the app owing to poorer digital literacy. Notwithstanding, the effectiveness of the Hypercare APK app can be influenced by technological, infrastructural, and user-related constraints.

In this study, a substantial proportion of the participants have relatively high educational status. Generally, high educational attainment enhances technical skills, information literacy, problem solving when the app crashes, faster onboarding and less drop-off at every stage of the use of the Hypercare APK app, encourages higher literacy use and overall experience that includes engagement, usability, trust and health outcome. This educational profile of the participants may have influenced their experiences with the Hypercare APK app. Most of the participants possessed tertiary-level education and were employed as skilled occupations, a characteristic that may be associated with higher level of digital literacy, greater familiarity with smartphone technologies and enhancing their ability to navigate, understand and engage with the Hypercare APK app seamlessly (24). Such high level of education demonstrated by the participants in this study may have increased their confidence of navigating the Hypercare APK app as well as their capacity to interpret health information delivered through the Hypercare APK app (25). These factors, taken together may have facilitated positive engagement with the app and contributed to the generally favourable perception reported in this study. In contrast, hypertensive patients with lower educational attainment or limited experience with digital technologies may encounter additional challenges related to Hypercare APK app navigation, interpretation of health information, trouble-shooting technical difficulties, and sustained engagement. Consequent upon this, caution is warranted when transferring these findings to Nigerian population with lower level of education, or limited digital access for they may encounter additional barriers related to the use of the Hypercare APK app. Future implementation efforts should consider simplified interfaces, multilingual options, additional user support, and digital literacy training to enhance accessibility across diverse populations of hypertensive patients in Nigeria.

### mHealth as a behavioural support tool

A central finding of this study is the critical role of reminder notifications in supporting the prompt to take medication. Participants consistently described reminders as the most valuable feature, highlighting their role as behavioural cues that prompt action amid competing daily demands. This aligns with behavioural theories of habit formation, where external prompts reinforce routine actions (26, 27). In this study, the Hypercare APK app compensated for forgetfulness, one of the most reported barriers to adherence, by embedding treatment prompts into patients' daily lives. Hinneh et al. (28) also opined that digital tools especially mobile health apps and SMS enhanced key hypertension self-management tasks in sub-Saharan African countries of Tanzania, South Africa, Kenya, Nigeria and Uganda.

### Enhancing patient engagement and self-management

The participants perceived that the Hypercare APK app facilitated active patient engagement, particularly through self-monitoring and access to health information. Participants reported increased awareness of their blood pressure and a greater sense of responsibility for their health (8). These findings suggest that mHealth interventions like Hypercare, in this instance, can shift patients from passive recipients of care to active participants in disease management (29). This is particularly important in hypertension, where long-term control depends heavily on sustained self-management behaviours.

### Importance of usability and simplicity

According to the participants, ease of use emerged as a key determinant of acceptability. Participants' positive experiences were largely attributed to the simplicity and intuitive design of the Hypercare APK app. This underscores the importance of user-centred design principles in digital health interventions. This ease of use of the app contributed significantly to initial acceptance and continued engagement. Apps, like this Hypercare APK app used in the present study, that are easy to navigate are more likely to achieve sustained engagement, particularly among populations with varying levels of digital literacy (13, 30).

### Structural and contextual barriers in low-resource settings

Despite its benefits, the use of Hypercare APK app was constrained by several context-specific barriers, including limited internet access, high data costs, unreliable electricity supply, and device incompatibility. These findings highlight a critical challenge in digital health implementation, the assumption of technological readiness in low-resource settings. Even, well-designed interventions may fail to achieve impact if underlying infrastructural limitations are not addressed. The findings here are similar to those of Aovare et al. (31) who opined unstable power and internet connectivity, increased data entry workload, limited patient access and digital literacy, and restricted roles for junior staff as barriers after exploring the Ghanian healthcare workers' experiences using the AfyaPro Connected Care app. These opinions were also similar to that reported by Aboye et al. (32) on a Sub-Sahara Africa Country, Ethiopia.

### Digital inequality and health equity

Language barriers and limited digital literacy further restricted access for some users, raising concerns about digital exclusion. The reliance on English-only content may inadvertently marginalise individuals with lower educational attainment or limited English proficiency. Addressing these disparities is essential to ensure that mHealth interventions do not widen existing health inequalities (1, 17). Features such as multilingual support and simplified interfaces are therefore not merely enhancements but essential components of equitable digital health design (33).

### Integration of Hypercare APK app into the health system

Participants strongly advocated integrating the application into routine healthcare delivery. This reflects a broader recognition that digital health tools are most effective when embedded within existing health systems rather than functioning as standalone interventions (34). Clinical endorsement, appointment integration, and institutional support may enhance trust, uptake, and sustained use. This integration also aligns with emerging models of patient-centred and digitally enabled care (29).

### Implications for practice and policy

The findings in the present investigation suggest that mHealth interventions like the Hypercare APK app, can play a significant role in improving hypertension management in low-resource settings. However, success depends on a number of factors including infrastructural barriers (data, electricity), enhancing usability and accessibility, ensuring cultural and linguistic relevance, and integrating digital tools into routine clinical care. Policymakers and healthcare institutions should prioritise investments in digital infrastructure and capacity-building to maximise the benefits of such interventions.

### Strengths and contributions

This study contributes to the limited qualitative evidence on mHealth use in sub-Saharan Africa by providing context-specific insights into user experiences with a locally developed app, Hypercare APK. The findings extend existing knowledge by highlighting the interplay between technological design and real-world contextual constraints.

This study demonstrates that the Hypercare APK app is a promising and contextually relevant digital tool for supporting hypertension self-management in a resource-limited setting. Participants perceived the Hypercare APK app as beneficial in prompting hypertensive medication use, enhancing self-monitoring practices, and increasing engagement with health information. These findings reinforce the growing role of mobile health interventions, like Hypercare APK app as accessible and scalable strategies for chronic disease management.

However, the effectiveness and sustainability of Hypercare APK app can be limited by contextual realities, including infrastructural limitations, digital literacy, language accessibility, and user trust. Persistent barriers such as unreliable internet connectivity, high cost of data, device incompatibility, and limited inclusivity in design highlight the need for context-sensitive adaptation of digital health solutions. Notwithstanding, embedding such apps into routine clinical care, supported by clinician engagement and institutional endorsement, may enhance uptake, trust, and long-term utilisation.

Future research should focus on large-scale implementation and long-term effectiveness across diverse populations to further establish the role of mHealth in improving cardiovascular health outcomes as well as large-scale, multi-centre studies incorporating both qualitative and quantitative approaches should be carried out.

### Limitations of the study

This study has several limitations that should be considered when interpreting the results.

(i) The perspectives of participants that discontinued the use of the Hypercare APK app, those that experienced difficulties with the app and those that declined participation were not captured. This might have resulted in selection biased and possible over-presentation of positive experiences in the present study.
(ii) Participants were recruited from a single tertiary healthcare institution in Ilorin, Nigeria, which may hamper transferability of the findings to other healthcare settings and populations
(iii) The relatively sample size predominantly consists of highly educated participants. Consequently, this will influence the outcome as individuals with lower educational attainment, limited health literacy, or less experience using digital technologies may not have been fully represented.
(iv) It is possible that some experience might have been forgotten or recalled differently over time because participants were requested to recall their experiences or challenges over the period of intervention
(v) Social desirability bias may have influenced participants' responses as some participants may have been inclined to provide favourable opinions about the Hypercare APK app

## Data Availability

The original contributions presented in the study are included in the article/Supplementary Material, further inquiries can be directed to the corresponding author.

## References

[1] Byiringo S Hinneh T Chepkorir J Tomiwa T Commodore-Mensah Y Marsteller J. Healthcare system barriers and facilitators to hypertension management in Ghana. Ann Global Health. (2024) 90(1):38. 10.5334/aogh.4246 PMC11229483 38978819

[2] Lu W Yuan J Liu Z Su Z Shen Y Li S. Worldwide trends in mortality for hypertension heart disease from 1990 to 2019 with projections to 2023: data from the global burden of disease 2019 study. Eur J Prev Cardiol. (2023). 10.1093/eurjpc/zwad262 37665956

[3] Zhou Z Ji Z Hou J Yang J Wu H Zhang L. Burden of hypertensive heart disease and its risk factors in east Asia, 1990-2021: findings from the global burden of disease study 2021. Glob Heart. (2025) 20(1):82. 10.5334/gh.1472 41019773 PMC12466111

[4] Ojo AE Ojji DB Grobbee DE Huffman MD Peters SAE. The burden of cardiovascular disease attributable to hypertension in Nigeria: a modelling study using summary-level data. Glob Heart. (2024) 19(1):50. 10.5334/gh.1332 38863890 PMC11166022

[5] World Health Organisation. Global Health Observatory – processed by our world in data. “Prevalence rate of hypertension in adults aged 30-79” [dataset]. World Health Organisation, “Global Health Observatory” [original data]. (2025). (Archived March 4, 2026). Available online at: https://archive.ourworldindata.org/20260304-094028/grapher/hypertension-adults-30-79.html (Accessed April 16, 2026).

[6] Okesina AA Habineza JC Mbazumutima R Mignonne U Mahirwe C Hakizimana S. Prevalence of undiagnosed hypertension and associated factors in the Ndera sector, Gasabo District, Rwanda: a cross-sectional study. BMC Public Health. (2024) 24:2495. 10.1186/s12889-024-19999-1 39272070 PMC11397070

[7] Kario K Okura A Hoshide S Mogi M. The WHO global report 2023 on hypertension warns of the emerging burden of hypertension worldwide and its treatment strategies. Hypertens Res. (2024) 47:1099–102. 10.1038/s4140-024-01622-w 38443614

[8] Hossain A Ahsan GU Hossain MZ. Medication adherence and blood pressure control in treated hypertensive patients: first follow-up findings from the PREDIcT-HTN study in northern Bangladesh. BMC Public Health. (2025) 25:250. 10.1186/s12889-025-21409-z 39838337 PMC11748311

[9] Zoccali C Mallamaci F Adamczak M De Oliveira R Massy Z Sarafidis P. Cardiovascular complications in chronic kidney disease: a review from the European Renal and Cardiovascular Medicine Working Group of the European Renal Association. Cardiovasc Res. (2023) 119:2017–32. 10.1093/cvr/cvad083 37249051 PMC10478756

[10] Ryabinina O Addo FO Thomford NE. Antihypertensive medication adherence and associated risk factors among adults with hypertension: a cross-sectional study in a teaching hospital, Ghana. BMC Cardiovasc Disord. (2026) 26:156. 10.1186/s12872-025-05410-3 41699474 PMC12908267

[11] Huda KR Chowhan SR Seervi D. Effectiveness of mobile health technology-enabled interventions to improve management and control of hypertension and diabetes in India- a systematic review. J Prev Med Rep. (2025) 54:103094. 10.1016/j.pmedr.2025.103094 PMC12104709 40421259

[12] Babatunde OA Ogundijo AD Afolayan OA Awosiku VO Aderohunmu OZ Oguntade SM. Mobile health technologies in the prevention and management of hypertension: a scoping review. Digit Health. (2024) 10:1–12. 10.1177/20552076241277172 PMC11363045 39221086

[13] Leuzzi G Recenti F Giardulli B Scafoglieri A Testa M. Exploring digital health: a qualitative digital study on adults’ experiences with health apps and wearables. Int J Qual Stud Health Well-Being. (2025) 20(1):2447096. 10.1080/17482631.2924.2447096 39726066 PMC11703456

[14] Tumuhimbise W Theuring S Kaggwa F Atukunda EC Rubaihayo J Atwine D. Enhancing the implementation and integration of mHealth interventions in resource-limited settings: a scoping review. Implement Sci. (2024) 19:72. 10.1186/s13012-024-01400-9 39402567 PMC11476919

[15] Alzghaibi H. Healthcare practitioners’ perceptions of mHealth application barriers: challenges to adoption and strategies for enhancing digital health integration. Healthcare. (2025) 13(5):494. 10.3390/healthcare13050494 40077056 PMC11899126

[16] De La Torre E Montane C Rubio O Sampietro-Colom L Camacho-Mahmud A Grau-Corral I. Integrating patient perspectives into the digital health technology readiness framework: Delphi study. J Med Internet Res. (2025) 27:e71600. 10.2196/71600 40835414 PMC12367350

[17] May S Muehlensiepen F Wengemuth E Seifert F Heinze M Bruch D. Benefits and barriers to mHealth in hypertension care: qualitative study with German health care professionals. J Med Internet Res. (2025) 12:e52544. 10.2196/52544 PMC11933770 40063928

[18] Onilude KA Esan TD Olajide AO Ogunmuyiwa AO Bamgboye TO Ramos CG. Exploring birth preparedness and complication readiness among expectant women: insights into perception, knowledge channels, and preparation practice in a resource-limited setting. Int J Afri Nurs Sci. (2026) 25:1011042. 10.1016/j.ijans.2026.101042

[19] Morrow R Rodriguez A King N. Colaizzi’s descriptive phenomenological method. Psychologist. (2015) 28(8):643–4.

[20] ATLAS.ti.Scientific Software Development GmbH B, Germany. Atlas.ti.software version 7 (2013).

[21] Naeem M Ozuem W Howell K Ranfagni S. A step-by-step process of thematic analysis to develop a conceptual model in qualitative research. Int J Qual Meth. (2023) 22:1–18. 10.1177/16094069231205789

[22] Sirwan KA. The pillars of trustworthiness in qualitative research. J Med Surg Public Health. (2024) 2:100051. 10.1016/j.glmedi.2024.100051

[23] Braun V Clarke V. Toward good practice in thematic analysis: avoiding common problems and be(com)ing a knowing researcher. Int J Transgend Health. (2022) 24(1):1–6. 10.1080/26895269.2022.2129597 36713144 PMC9879167

[24] Muehlensipen F May S Seifert F Wengemuth E Johannsen O Middeke M. User profiles and engagement in a hypertension self-management app: cross-sectional survey. J Med Internet Res. (2026) 28:e83075. 10.2196/83075 41671553 PMC12893642

[25] Anyaobi G Echedom AU. Educational qualification and experience as correlates to digital literacy and information access among library and information science educators in universities in south-south Nigeria. J Appl Inf Sci Technol. (2025) 18(1):64–80. 10.70118/jaist.202501801.8

[26] Arshed M Kiran M Umar MF Samkari JA Hassan SMU Qamer S. Prevalence and associated factors of adherence to antihypertensive medication: a nationwide cross-sectional study. BMC Public Health. (2025) 25(1):2364. 10.1186/s12889-025-21456-6 40610935 PMC12224808

[27] Wong W Nguyen TV Nguyen VT Ngo KTT Nguyen TN. Forgetfulness to take antihypertensive medications and poor blood pressure control in older adults with type 2 diabetes and hypertension in Vietnam. J Clin Hypertens. (2025) 27(8):e70105. 10.1111/jch.70105 PMC12317329 40751466

[28] Hinneh T Mensah B Kwanin C Okonkwo C Byiringiro S. Digital health tools in hypertension management in sub-Saharan Africa: a scoping review of barriers and facilitators of adoption into mainstream healthcare. BMJ Health Care Inform. (2025) 32(1):e101261. 10.1136/bmjhci-2024-101261 41073105 PMC12517033

[29] Gulati M Moore M Cibotti-Sun M. High blood pressure guideline-at-a-glance. J Am Coll Cardiol. (2025) 86(18):1560–6. 10.1016/j.jacc.2025.07.010 40817900

[30] Still CH Alnomasy N Irani L Gary FA. Barriers to and facilitators of using mHealth technology among African-Americans living with hypertension. J Nat Black Nurses Assoc. (2022) 33(10):1–7. PMC13247363 38564485

[31] Aovare P Beune E Chilunga FP Moens N Mollvan Charante EP Agyemang C. The acceptability, adoption, and feasibility of mobile health interventions for diabetes and hypertension care among Ghanaian healthcare workers. PEC Innov. (2026) 8:100456. 10.1016/j.pecinn.2026.100456 41646125 PMC12870867

[32] Aboye GT Simegn GL Aerts JM. Assessment of the barriers and enablers of the use of mHealth systems in sub-Saharan Africa according to the perceptions of patients, physicians, and health care executives in Ethiopia: qualitative study. J Med Internet Res. (2025) 26:e50337. 10.2196/50337 PMC11007608 38536231

[33] Whitehead L Talevski J Fatehi F Beauchamp A. Barriers to and facilitators of digital health among culturally and linguistically diverse populations: qualitative systematic review. J Med Internet Res. (2022) 25:1–19. 10.2196/42719 PMC10015358 36853742

[34] Dweik R Ajaj R Kotb R Halabi D Sadier N Sarsour H. Opportunities and challenges in leveraging digital technology for mental health system strengthening: a systematic review to inform interventions in the United Arab Emirates. BMC Public Health. (2024) 24:1–11. 10.1186/s12889-024-19980-y 39334131 PMC11429924

