# Supplementary material for: Barriers and facilitators to the use of Hypercare APK app in hypertension management: a qualitative inquiry into highly educated participants’ experiences in Ilorin, Nigeria
Source: Front Digit Health. 2026 Jul 31;8:1891831. doi: 10.3389/fdgth.2026.1891831 (PMC13473258; doi:10.3389/fdgth.2026.1891831)
Supplement: Supplementary file 1 [file Supplementaryfile1.docx]

**HyperCare APK**

Mobile Health Monitoring Application

Technical Overview Report

Prepared: June 2026

# **1. Application Overview**

HyperCare is an Android mobile application designed to support the monitoring and management of patients with hypertension and related cardiovascular conditions. The application facilitates direct communication between patients and their assigned healthcare providers (nurses), enabling continuous remote health monitoring outside the clinical setting.

## **Application Identity**

| **Property** | **Detail** |
| --- | --- |
| Application Name | HyperCare |
| Platform | Android (Mobile) |
| Package Identifier | com.example.hypercare |
| Target Users | Patients (hypertension) and Nurses/Healthcare Providers |
| Backend Infrastructure | Firebase Realtime Database & Firebase Authentication |
| Data Transmission | Encrypted real-time cloud synchronisation (Google Firebase) |

# **2. User Roles**

The application supports two distinct user roles, each with a tailored interface and corresponding access privileges:

| **Role** | **Description** | **Primary Responsibilities** |
| --- | --- | --- |
| Patient | Registered patient under hypertensive care | Log vitals, view health records, communicate with assigned nurse, receive medication reminders |
| Nurse / Healthcare Provider | Assigned clinical staff member | Monitor patient vitals, manage appointments, communicate with patients, generate health record PDFs |

# **3. Clinical Features**

## **3.1 Vital Signs Monitoring**

Patients can record and track the following physiological parameters within the application. Values are stored with accurate timestamps using Network Time Protocol (NTP) synchronisation to ensure data integrity:

| **Parameter** | **Unit** | **Recorded By** |
| --- | --- | --- |
| Blood Pressure | mmHg | Patient (self-reported) |
| Heart Rate / Pulse | bpm (beats per minute) | Patient (self-reported) |
| Body Weight | kg (kilograms) | Patient (self-reported) |
| Height | m (metres) | Patient (profile setup) |
| Age | Years | Patient (profile setup) |

## **3.2 Longitudinal Health Records**

All vital sign entries are stored chronologically with NTP-accurate timestamps, creating a longitudinal health record for each patient. Nurses can access these records from the patient management dashboard. A built-in PDF export function allows health records to be printed or saved for clinical documentation and handover purposes.

## **3.3 Patient Dashboard (Nurse View)**

The nurse-facing dashboard presents a real-time overview of all registered patients, displaying their most recent recorded values for blood pressure, heart rate, and body weight. This allows the healthcare provider to quickly identify patients who may require attention without needing to open individual records.

## **3.4 Appointment Scheduling**

Nurses can schedule appointments for individual patients using a built-in date and time picker. Scheduled appointments are stored in the cloud and are visible to the patient. Key appointment features include:

- Nurse-initiated appointment creation with precise date and time selection
- Patient view shows the next upcoming active appointment with a live countdown timer (hours and minutes remaining)
- Appointment status tracking (active/completed)
- Nurse dashboard displays the total number of appointments and those scheduled for the current day

## **3.5 Medication Reminders**

The application delivers automated push notifications to support medication adherence. Reminders are scheduled daily at two fixed times and persist across device restarts through a Boot Receiver mechanism:

| **Reminder** | **Time** | **Notification Message** |
| --- | --- | --- |
| Morning Dose | 8:00 AM daily | "Good morning! It's time to take your drugs." |
| Evening Dose | 6:00 PM daily | "Evening reminder. Please take your drugs." |

## **3.6 Patient–Nurse Messaging**

A real-time messaging system allows direct two-way communication between a patient and their assigned nurse. Messages are delivered instantly via Firebase and the application sends push notifications for incoming messages when the chat window is not in focus. Each patient is assigned to a specific nurse, maintaining a one-to-one care relationship.

## **3.7 Health Tips**

The patient home screen displays randomly selected health education tips sourced dynamically from the cloud database. Tips are refreshed periodically (every 10 minutes) to provide varied health guidance relevant to hypertension management and general wellbeing.

# **4. Data Management and Security**

All patient data is stored in Google Firebase Realtime Database, a cloud-based NoSQL database with industry-standard encryption in transit (TLS). User authentication is managed through Firebase Authentication using email and password credentials. Each patient record is uniquely identified by a Firebase-generated UID.

Access to patient data is role-differentiated: patients can only view and update their own records, while nurses have read access to all registered patients' vitals through the dashboard. Health update timestamps are fetched from an external NTP server (time.google.com) to ensure the accuracy and tamper-resistance of clinical timestamps.

# **5. Feature Summary**

| **Feature** | **Available** |
| --- | --- |
| Blood pressure logging | Yes |
| Heart rate / pulse logging | Yes |
| Body weight logging | Yes |
| Timestamped health history | Yes |
| Health record PDF export | Yes |
| Appointment scheduling (nurse) | Yes |
| Appointment countdown (patient) | Yes |
| Daily medication reminders (x2) | Yes |
| Real-time patient–nurse messaging | Yes |
| Push notifications for messages | Yes |
| Health tips for patients | Yes |
| Patient list dashboard (nurse) | Yes |
| Secure cloud data storage | Yes |
| Role-based access (patient/nurse) | Yes |

# **6. Note on Application Scope**

HyperCare is a patient-facing monitoring and communication aid and does not replace clinical assessment or professional medical advice. Vital sign data entered in the application is self-reported by the patient and should be interpreted in conjunction with clinical evaluation. The application is intended to supplement, not substitute, standard care pathways for hypertensive patients.

**Screen shots of Some Daily Healthy Tips from Hypercare APK app**

**The interface of the Hypercare APK app**

**Hypercare APK app**
