# Supplementary material for: Barriers and facilitators to the use of Hypercare APK app in hypertension management: a qualitative inquiry into highly educated participants’ experiences in Ilorin, Nigeria
Source: Front Digit Health. 2026 Jul 31;8:1891831. doi: 10.3389/fdgth.2026.1891831 (PMC13473258; doi:10.3389/fdgth.2026.1891831)
Supplement: Supplementary file 2 [file Supplementaryfile2.doc]

# **SUPPLEMENTARY 2**

**Interview Guide to assess Barriers and Facilitators to the use of a Mobile Health (mHealth) intervention among Hypertensive Patients**

Study Title:

Impact of a Mobile Health Intervention on Adherence to Treatment Regimens among Hypertensive Patients in Tertiary Hospitals in Ilorin: A Non-Randomized Controlled Trial

Participant introduction and set-up

The researcher introduces self to the participant.

The researcher explains the following to the participant:

The purpose of the interview is to have an in-depth understanding of experiences, challenges, and supportive factors influencing the use of mobile health (mHealth) applications to manage hypertension.

The interview may last up to 20 minutes, but it can be shorter if you don’t have that much time.

Format: Semi-structured; using probes to encourage detailed responses

The interview will be recorded, and we will write up what you said word-for-word. When writing this up, we will remove anything that identifies you (e.g., name), where you live, or anyone else you mention, so it will all be anonymous. Also, everything we talk about here will be confidential.

We can take a break at any time you like. Please just let me know, and I can pause the recording.

Researcher obtains informed consent from the participant (Check consent form has been completed).

Researcher thanks the participant (Thank you for completing/returning your consent form).

The researcher asks the participant if they have any questions about any aspect of the interview.

If you are willing to proceed, I will start the interview recording now.

**Data collection**

**Section A:** Participant’s socio-demographic profile

Prompts:

Can you tell me a bit about yourself (e.g., age, gender)?

What is your level of education?

How many years have you been managing hypertension?

**Section B**: Experience with the use of the Mobile App

Prompts:

How did you use the mobile app during the period of study?

Which features did you use most (reminders, monitoring, daily education tips)?

How useful was the mobile app in managing your blood pressure?

Did it help with compliance and lifestyle modifications, or monitor your progress?

**Section C**: Facilitators

Which features of the mobile app made it easy or motivated you to use it?

Prompts:

Notifications, drug reminders, easy interface, and daily tips.

Are there any instances or situations when the app is most useful?

Prompts:

Morning routine, taking medications, tracking diet, and BP.

**Section D**: Barriers

What obstacles or difficulties did you face while using the mobile app?

Prompts:

Technical issues, app navigation, cost (data) connectivity, and understanding messages

Were there any times you did not use the mobile app as recommended? Why?

Prompts:

Busy schedule. Forgot, reminder alert is disturbing, and lack of motivation

**Section E:** Recommendations

In your opinion, what could make the app easier or more helpful for an individual managing blood pressure?

Prompts:

Simple instructions, more reminders, and additional educational content

Would you like to see this incorporated into hypertension treatment in healthcare facilities in Nigeria?

**Closing Question:** Is there anything else you would like to share about your experiences using the mobile app?

Prompts:

Suggestions, or concerns

**Concluding**

The researcher thanks participants for their time and for the information shared

Participants are once again reassured of the confidentiality of their information.

The researcher will close the session and label the recording.
